# Prevalence and Genetic Diversity of *Blastocystis* sp. in Humans, Dogs and Cats in Gabon: A One Health Perspective

**DOI:** 10.1111/zph.70044

**Published:** 2026-02-19

**Authors:** P. Makouloutou‐Nzassi, N. M. Longo‐Pendy, G. Ndong Atome, N. Mpassy, F. Bangueboussa, N. C. Atiga, A. P. Okouga, N. B. N'dilimabaka, S. L. Oyegue‐Liabagui, J. B. Lekana‐Douki, L. Boundenga

**Affiliations:** ^1^ Département de Biologie et Ecologie Animale Institut de Recherche en Ecologie Tropicale (IRET/CENAREST) Libreville Gabon; ^2^ Unité de Recherche en Ecologie de la Santé, Centre Interdisciplinaire de Recherches Médicales de Franceville Franceville Gabon; ^3^ Department of Biochimie, Faculty of Science Masuku University of Science and Technology (USTM) Franceville Gabon; ^4^ Unit of Evolution, Epidemiology and Parasite Resistance (UNEEREP) Franceville Interdisciplinary Center for Medical Research (CIRMF) Franceville Gabon; ^5^ Department of Biology, Faculty of Science Masuku University of Science and Technology (USTM) Franceville Gabon; ^6^ Central African Regional Doctoral School in Tropical Infectiology (ECODRAC) Franceville Gabon; ^7^ Department of Parasitology‐Mycology University of Health Sciences (USS) Libreville Gabon; ^8^ Department of Anthropology Durham University Durham UK

**Keywords:** *Blastocystis* sp., cats, dogs, Gabon, humans, One Health, prevalence, subtypes

## Abstract

**Introduction:**

*Blastocystis* sp. is a cosmopolitan protist that colonises the gastrointestinal tract of mammals, including humans, dogs and cats. Although its pathogenicity remains debated, *Blastocystis* sp. has been linked to gastrointestinal symptoms and alterations in the gut microbiota. In Gabon, most epidemiological data on *Blastocystis* sp. rely on microscopy, which may underestimate true prevalence. Moreover, molecular studies remain scarce, and none have investigated the parasite's presence in domestic animals. This study aimed to estimate the prevalence and subtype (ST) distribution of *Blastocystis* sp. in humans, dogs and cats across three provinces of Gabon, within a One Health framework.

**Methods:**

Between June 2021 and November 2022, a total of 182 faecal samples were collected: 127 from children in the Mulundu department (Ogooué‐Lolo), 55 from both dogs and cats in Libreville (Estuaire) and Franceville (Haut‐Ogooué). Samples were screened for the presence of *Blastocystis* sp. using conventional PCR targeting the SSU rRNA gene, positive isolates were subtyped.

**Results:**

*Blastocystis* sp. was detected in 58.3% of human samples, with a significant association between infection and place of residence (*p* = 0.02, fisher's exact test). Among animals, the parasite was found in 18.2% (8/44) of dogs and 36.4% (4/11) of cats. Subtyping revealed ST1 and ST3 in humans, while only ST1 was found in dogs. No subtypes were resolved in cats. These findings confirm the high circulation of *Blastocystis* sp. in both humans and domestic animals in Gabon and highlight the potential for zoonotic transmission.

**Conclusion:**

This study provides the first molecular evidence of *Blastocystis* sp. infection in animals in Gabon and underscores the need for integrated surveillance strategies to better understand its epidemiology.

## Summary

- *Blastocystis* sp. is a unicellular eukaryote commonly found in the intestinal tract of humans and other animals. More than 40 subtypes have been identified globally.
- *Blastocystis* sp. have been found in over half of the children tested but also in domestic animals like dogs and cats.
- This study provides for the first time valuable data on the prevalence and subtype of *Blastocystis* sp. that circulate among humans and domestic animals in Gabon.

**Abbreviations**
NHPs: non‐human primates
NJ: neighbour‐joining
ST: subtype

## 1 Introduction

Emerging eukaryotic pathogens pose a growing public health concern worldwide. Among these, *Blastocystis* sp., a protist that inhabits the gastrointestinal tract of various hosts, including humans (Aung et al. 2025; Boorom et al. 2008) and domestic animals such as dogs and cats (Shams, Asghari, et al. 2022). This widely distributed microorganism exhibits four major life stages (granular, vacuolar, amoeboid and cystic) and is frequently detected in both symptomatic and asymptomatic individuals (Fusaro et al. 2024; de Melo et al. 2020). While the pathogenicity of *Blastocystis* sp. remains debated, infections have been associated with gastrointestinal symptoms such as diarrhoea, abdominal pain and cramps, as well as chronic conditions like irritable bowel syndrome (IBS) and urticaria, particularly in immunocompromised patients (Pessarelli et al. 2022). Beyond its potential clinical relevance, *Blastocystis* sp. also interacts with the gut microbiota. Some studies suggest it enhances microbial diversity and may contribute to a healthier gut environment (Audebert et al. 2016; Even et al. 2021; Tito et al. 2019), while others report a disruptive impact on microbial balance (Yason et al. 2019). Transmission of *Blastocystis* sp. primarily occurs via the faecal‐oral route through ingestion of infective cysts (Rudzińska and Sikorska 2023). Human‐to‐human, animal‐to‐human and reverse zoonotic transmission have also been documented, often via contact with infected individuals or through consumption of contaminated food or water (Pérez et al. 2020). The global prevalence of *Blastocystis* sp. varies widely, from 15% to 25% in industrialised nations to over 50% in developing regions, particularly in Africa (Alfellani, Taner‐Mulla, et al. 2013; El Safadi et al. 2014). Molecular analysis of the small subunit ribosomal RNA (SSU‐rRNA) gene has identified 44 subtypes (ST1–ST44) to date (Aydemir et al. 2024; Jinatham et al. 2024), though the classification of ST18, ST20 and ST22 remains controversial (Matovelle et al. 2024). ST1‐ST9 and ST12 have been isolated from humans, while the others are primarily animal‐associated (Wang et al. 2024). Among human infections, ST1–ST4 account for over 90% of reported cases (Popruk et al. 2021; Ryckman et al. 2024). In dogs, ST1–ST8, ST10, ST23 and ST24 have been detected with ST3 being the most prevalent (Shams, Shamsi, et al. 2022). Zoonotic transmission is mainly attributed to ST1–9 and ST12, whereas ST10–11 and ST13–17 are found predominantly in non‐human hosts.

In Gabon, the prevalence of *Blastocystis* sp. among symptomatic individuals rose dramatically from 0.7% in 2004 to 45.6% in 2014 (M'Bondoukwé et al. 2023). High infection rates have also been reported in both rural (41.6%) and urban slum populations (48.6%), likely driven by poor sanitation, limited access to clean water and close human‐animal interactions (Deng et al. 2021; Lengongo et al. 2020; M'bondoukwé et al. 2016, 2018). However, the majority of these studies relied on microscopy, which may underestimate actual prevalence. Only two studies have employed molecular techniques to detect *Blastocystis* sp. in human faecal samples in Gabon: Oyegue‐Liabagui et al. (2020) failed to detect the parasite, whereas Ndong Mba et al. (2025) reported a molecular prevalence of 23.43%. To date, no molecular data were found on *Blastocystis* sp. in animals in our review on Gabonese studies (Boundenga et al. 2021; Dibakou et al. 2021), leaving significant gaps in our understanding of zoonotic transmission routes and genetic diversity.

Therefore, this study aims to estimate the prevalence and subtype distribution of *Blastocystis* sp. in humans, dogs and cats across Gabon. It also seeks to evaluate the potential risk of zoonotic transmission within a One Health framework. To our knowledge, this is the first comprehensive investigation in Gabon simultaneously addressing the occurrence and molecular diversity of *Blastocystis* sp. in both humans and companion animals. By filling this gap, the study contributes to a better understanding of *Blastocystis* sp. epidemiology and its implications for public and veterinary health in this region.

## 2 Materials and Methods

### 2.1 Study Design and Sample Collection

This cross‐sectional study was conducted in the Lastourville region (0°49′ S, 12°42′ E) of the Ogooué‐Lolo province in Gabon, a rural area with an equatorial climate characterised by a short dry season from June 15 to September 15 and a long rainy season from September 15 to June 15. Samples from humans and animals were collected from various geographic areas to facilitate an initial, comprehensive assessment of *Blastocystis* sp. circulation across Gabon. The study population included children aged 3–17 years who were enrolled in primary schools in the region.

### 2.2 School and Participant Selection

A two‐stage random sampling method was implemented. In the first stage, a comprehensive list of all public primary schools in Lastourville was obtained from the local education authority. Schools were assigned numbers, and four were randomly selected using a computer‐generated list. In the second stage, eligible children were randomly chosen from class rosters within each selected school. Participation was voluntary; only children who had resided in the study area for at least three months and whose parents or legal guardians provided written informed consent were included in the study.

### 2.3 Inclusion and Exclusion Criteria

Children were eligible if they were between the ages of 3 and 17 and attending one of the selected schools. Exclusion criteria included (i) the recent use of antiparasitic drugs (within the previous month), (ii) current acute gastrointestinal symptoms (such as diarrhoea or vomiting) and (iii) inability to provide a stool sample on the day of collection.

### 2.4 Sample Size Considerations

The final sample comprised 127 children. No formal power calculation was conducted; instead, the sample size was determined based on logistical feasibility, the number of children available during the study period and the resources available for sample processing. This approach aligns with exploratory cross‐sectional parasitological surveys in similar settings.

### 2.5 Community Sensitisation and Data Collection Procedures

Prior to data collection, the research team held community sensitisation meetings with local authorities, teachers and parents to outline the study's objectives, procedures and benefits. Each participant underwent a brief interview and medical examination. Stool samples were collected in sterile, pre‐labelled containers, stored at 4°C and transported daily to a field laboratory for analysis.

### 2.6 Animal Sample Collection

Faecal samples from animals were collected in the cities of Libreville (Estuaire province) and Franceville (Haut‐Ogooué province). The sampled animals (*n* = 55; dogs and cats) were roadkill carcasses identified on public roads, collected with formal authorisation from municipal authorities. Roadkill sampling was selected as provides a non‐invasive and ethically acceptable method for specimen collection, commonly used in the surveillance of wildlife and domestic animal diseases. Based on field observations and consultations with local veterinarians, most carcasses were from free‐roaming or semi‐owned animals, which are likely to interact with the environment and potentially serve as reservoirs for zoonotic parasites. To minimise contamination, carcasses were collected shortly after observation (generally within a few hours), stored in sealed body bags and transported to the laboratory, where faecal material was aseptically retrieved from the gastrointestinal tract. However, we acknowledge that roadkill sampling may introduce biases related to scavenging or environmental exposure; these limitations will be discussed further.

### 2.7 DNA Extraction and Molecular Detection

Genomic DNA was extracted from 200 mg of each faecal samples using the ROBOKLON stool DNA kit (EURx Ltd. 80–297 Gdansk Poland ul. Przyrodnikow 3, NIP 957‐07‐05‐191 KRS 0000202039, www.eurx.com.pl), following the manufacturer's instructions. Detection of *Blastocystis* sp. was performed using conventional polymerase chain reaction (PCR) targeting a 350 bp fragment of the small subunit ribosomal RNA (SSU rRNA) gene (Scicluna et al. 2006). The amplification used the *Blastocystis*‐specific reverse primer BhRDr (5′‐GAGCTTTT TAACTGCAACAACG‐3′) and the broad‐range eukaryote forward primer RD5 (5′‐ATCTGGTT GATCCTGCCAGT‐3′). Each PCR reaction had a final volume of 25 μL, which included 12.5 μL of GoTaq G2 Hot Start Colorless Master Mix (Promega), 0.75 μL of each primer (10 μM), 4 μL of DNA template and 7 μL of nuclease‐free water. Amplification was conducted under the following conditions: an initial denaturation at 95°C for 2 min, followed by 35 cycles consisting of 30 s of denaturation at 95°C, 30 s of annealing at 58°C and 30 s of extension at 72°C, with a final extension at 72°C for 5 min. *Blastocystis* sp.‐positive DNA and nuclease‐free water controls were included in each run. PCR products were visualised on 1.5% agarose gels prepared in TAE buffer and stained with GelRed (Biotium Inc., Fremont, CA, USA) following electrophoresis.

### 2.8 Sequencing and Phylogenetic Analysis

To determine the molecular identity and subtype distribution of *Blastocystis* sp., phylogenetic analysis was performed based on partial sequences of the 18S rRNA gene. Reference sequences representing known *Blastocystis* sp. subtypes were retrieved from GenBank and used for comparison. Multiple sequence alignments were performed using ClustalW (Hall 1999) in BioEdit v.7.0.9.0.

A maximum likelihood (ML) phylogenetic tree was constructed using a 350‐base pair fragment of the 18S rRNA gene. The best‐fit nucleotide substitution model, identified using ModelTest (Posada and Crandall 1998) under the Akaike information Criterion (AIC), was the general time‐reversible model with gamma‐distributed rate variation (GTR+I). Trees construction and bootstrap analysis (100 replicates) were conducted using the online PhyML2 platform, applying nearest‐neighbour interchange (NNI), subtree pruning and regrafting (SPR) and branch swapping algorithms (Guindon et al. 2010). Bootstrap values were used to assess the robustness of the inferred phylogenetic relationships.

### 2.9 Statistical Analysis

Data analysis was performed using Microsoft Excel 2016 (Microsoft Corp., Washington, USA) and R software version 4.3.2 (R Foundation for Statistical Computing, Vienna, Austria). Categorical variables are reported as frequencies, and prevalence estimated are accompanied by corresponding *p*‐values. Fisher's exact test was used to compare the frequency of *Blastocystis* sp. carriage among humans, dogs and cats based on molecular detection results. A *p*‐value of less than 0.05 was considered statistically significant.

## 3 Results and Discussion

The PCR screening results are summarised in Tables 1 and 2. The current study found a high prevalence of *Blastocystis* sp. among children in Lastourville, Gabon, at 58.3%. Additionally, the parasite was detected in dogs (18.2%) and cats (36.4%). However, caution is needed when interpreting the infection rates in cats due to the small sample size (*n* = 11), which limits the reliability of the prevalence estimates.

**TABLE 1:** Prevalence of *Blastocystis* sp. in the human population.

Features	Positives *n*+ (%)	Negatives *n*− (%)	*p‐value*	Test
Gender				
Women	35 (50.7)	34 (49.3)	0.089	Chi^2^
Men	39 (67.2)	19 (33.8)		
Slices of ages (years)				
5–10	33 (57.9)	24 (42.1)		
11–16	34 (57.6)	25 (42.4)	1	Chi^2^
Residences				
Bakoussou	1 (50.0)	1 (50.0)		
Ceb Bambidi	17 (47.2)	19 (52.8)		
Lastourville	34 (54.8)	28 (45.2)	0.02*	Fisher
Ndangui district	19 (86.4)	3 (13.6)		
Ndekabalandji	3 (60.0)	2 (40.0)		

Abbreviations: (%), percentage; *n*−, negative; *n*+, case positive.

^*^ Test significant.

**TABLE 2:** Prevalence of *Blastocystis* in animal population (dogs and cats).

Features	Positive *n*+ (%)	Negative *n*− (%)	*p‐value*	Test
Species				
Dogs	8 (18.2)	36 (81.8)		
Cats	4 (36.4)	7 (63.4)	0.3693	Chi^2^
Gender				
Male	6 (23.01)	20 (76.9)		
Female	5 (25.0)	15 (75.0)	0.688	Fisher
N/A	1 (11.1)	8 (88.9)		
Locality				
Franceville	10 (23.8)	32 (76.2)	0.7	Fisher
Libreville	2 (15.4)	11 (84.6)		
Breed				
Exotic	1 (33.3)	2 (66.7)		
Local	9 (20.0)	36 (80.0)	0.5326	Fisher
N/A	2 (28.6)	5 (71.4)		

Abbreviations: (%), percentage; *n*−, negative; *n*+, case positive.

No significant differences were found in infection rates between boys (67.2%) and girls (50.7%) (*p* = 0.089), nor across age groups (57.9% in 5–10‐year‐olds vs. 57.6% in 11–16‐year‐olds). However, a significant geographic variation was observed (*p* < 0.05), with a substantially highest prevalence recorded in the peri‐urban district of Ndangui (86.4%) compared to the urban area of Lastourville (54.8%). This difference is likely linked to disparities in sanitation infrastructure and access to clean water.

Among domestic animals, *Blastocystis* sp. was detected in 18.2% (8/44) of dogs and 36.4% (4/11) of cats. Infection rates were higher in Franceville (23.8%) than in Libreville (15.4%) and more frequent in local breeds. Although exotic breeds showed a higher percentage of infection (33%), this difference was not statistically significant.

**FIGURE 1:**
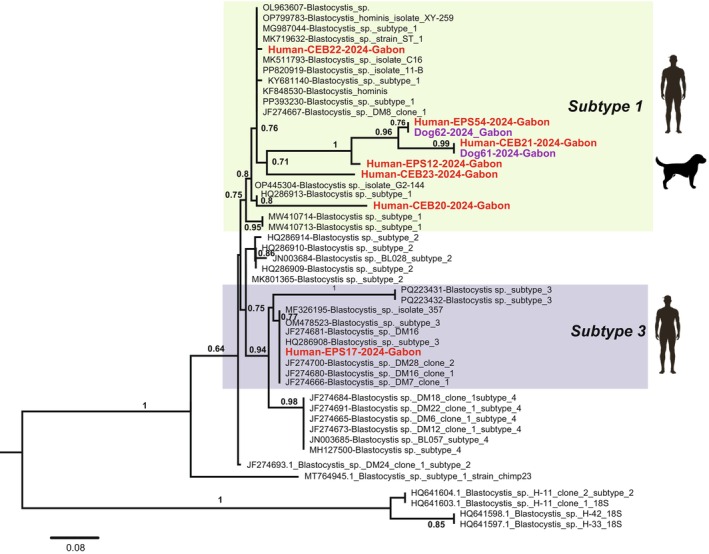
Phylogenetic tree illustrating the relationships between *Blastocystis* sp. sequences obtained in this study and reference sequences of different subtypes retrieved from GenBank.

Subtype analysis revealed the presence of subtype 1 (ST1) and subtype 3 (ST3) in human samples, while only ST1 was detected in dogs (Table 3). This analysis focused on a ~350 bp fragment of the SSU rRNA gene, which, while adequate for initial identification, can limit resolution between closely related subtypes and may underestimate diversity. Longer sequences, such as the 600 bp barcoding region, are generally preferred for definitive subtype assignments. To account for potential mixed infections, PCR chromatograms were carefully examined for double peaks, and samples with ambiguous signals were excluded from phylogenetic analysis. Although the detection of the shared subtype (ST1) suggests potential cross‐species transmission, the lack of paired human‐dog samples from the same households limits definitive conclusion about the transmission routes. No STs were identified in feline samples, possibly due to low DNA quality or quantity. Phylogenetic analysis (Figure 1) confirmed the identity of the STs, clustering them with reference sequences. While a higher number of bootstrap replicates is typically recommended for greater robustness, the strong support values (> 90%) for the major ST1 and ST3 clades instill confidence in these primary assignments. These findings align with studies from other regions from Africa, where ST1–ST3 are predominate, although they contrast with reports from other regions (Alfellani, Stensvold, et al. 2013).

**TABLE 3:** *Blastocystis* STs distribution among humans and dogs in Gabon.

Sample ID	Host	Subtype	Locality
12 EPS	Human	ST1	Setrag/LTV Station
20 CEB	Human	ST1	CEB Mbambidi
22 CEB	Human	ST1	CEB Mbambidi
23 CEB	Human	ST1	CEB Mbambidi
14 EPS	Human	ST3	Setrag/LTV Station
17 EPS	Human	ST3	Setrag/LTV Station
24 EPS	Human	ST1	Setrag/LTV Station
Dog_61	Dog	ST1	FCV
Dog_62	Dog	ST1	FCV

*Note:* The table presents the *Blastocystis* subtypes (ST) identified in the study population samples, along with the corresponding locality of origine.

This study provides the first molecular evidence of *Blastocystis* sp. infection in humans and domestic animals in Gabon, establishing the country as an endemic region. The human prevalence is similar to that reported in Côte d'Ivoire (58.2%) (D'Alfonso et al. 2017), but lower than in Cameroon (88.2%) (Lokmer et al. 2019), with these disparities potentially reflecting regional differences in sanitation, water quality and diagnostic methods.

The absence of sex‐related differences in infection aligns with findings from Nigeria (Poulsen et al. 2016) and Turkey (Beyhan et al. 2015). Similarly, the lack of age‐related contrast with reports of higher prevalence in younger children from other regions (Poulsen et al. 2016; Delshad et al. 2020), which may be attributed to uniform environmental exposure or collective play behaviour in this cohort (Benouis et al. 2013). The pronounced geographic variation, with higher prevalence in peri‐urban areas, mirrors observations from Senegal (El Safadi et al. 2014) and Colombia (Osorio‐Pulgarin et al. 2021), where inadequate sanitation is a risk factors for *Blastocystis* sp. transmission.

The canine infection rate (18.2%) is lower than that reported in Colombia (87.5%) (Higuera et al. 2021) but higher than rates in Turkey (1.5%) (Ayan et al. 2024) and France (3.4%) (Osman et al. 2015). Similarly, the prevalence in cats (34.8%) exceeds rates from Egypt (2.6%) (Naguib et al. 2022) and France (0%) (Cian et al. 2017) but is lower than in Australia (63.7%) (Shams, Asghari, et al. 2022). These global discrepancies likely reflect difference in climate, environmental hygiene and host susceptibility.

The exclusive detection of ST3 in humans raises the possibility of anthroponotic (human‐to‐human) transmission, while the presence of ST1 in both humans and dogs suggests a potential zoonotic cycle, supported by global data indicating ST1 as a frequently shared subtype (Alfellani, Taner‐Mulla, et al. 2013; Mahdavi et al. 2022; Mohammadpour et al. 2020; Roberts et al. 2013; Shams, Shamsi, et al. 2022; Tan et al. 2013). While we could isolate only one ST (ST1) in our dog's samples, Mahdavi et al. isolated three STs (ST1, ST2 and ST3) in their study on pet dogs in Shiraz, southwestern Iran (Mahdavi et al. 2022). The reasons for this discrepancy are discussed below.

A key limitation of this study was the low sequencing success, with only 8 human isolates and a few canine isolates sequenced, despite a larger number of PCR‐positive samples. This low success rate was likely due to factors such as low DNA concentration, PCR inhibitors, or the use of a short SSU rRNA fragment. As previously mentioned, this short fragment may have restricted subtypes resolution, resulting in an underestimated of subtype diversity. Furthermore, the small sample sizes for cats (*n* = 11) and the lack of paired human‐animal and environmental samples preclude firm conclusions on transmission dynamics. Lastly, the phylogenetic analysis was constrained by a lower number of bootstrap replicates (100) than is typically recommended.

Overall, our findings clearly demonstrate the presence of *Blastocystis* sp. in both humans and companion animals in Gabon. Although the subtype distribution suggests the possibility of both anthroponotic and zoonotic transmission, the exact transmission routes have yet to be confirmed due to the limitations previously mentioned.

## 4 Conclusion

This study provides the first molecular evidence of *Blastocystis* sp. in both humans and companion animals in Gabon. It reveals the high prevalence of the parasite in humans and identifies the presence of ST1 in both humans and dogs. While these findings suggest the possibility of cross‐species transmission, they do not confirm it. The findings also indicate that environmental exposure may play a role, particularly in peri‐urban areas.

Further studies using larger sample sizes, paired human–animal sampling, environmental surveillance and more discriminating molecular markers such as MLST are needed to clarify transmission dynamics and identify actual reservoirs. Employing a comprehensive One Health approach will be essential for unravelling the ecological complexity of *Blastocystis* sp. and informing public health strategies in Gabon and similar settings.

## Author Contributions

Conceptualisation: P. Makouloutou‐Nzassi, S.L. Oyegue‐Liabagui and N.B. N'dilimabaka. Methodology: P. Makouloutou‐Nzassi, N.M. Longo‐Pendy, F. Bangueboussa and N. Mpassy. Software: P. Makouloutou‐Nzassi and N.M. Longo‐Pendy. Validation: P. Makouloutou‐Nzassi, S.L. Oyegue‐Liabagui and N.B. N'dilimabaka. Formal analysis: P. Makouloutou‐Nzassi and N.M. Longo‐Pendy. Investigation: P. Makouloutou‐Nzassi, N.C. Atiga, A.P. Okouga and F. Bangueboussa. Resources: J.B. Lekana‐Douki, N.B. N'dilimabaka and S.L. Oyegue‐Liabagui. Data curation: P. Makouloutou‐Nzassi. Writing – original draft preparation: P. Makouloutou‐Nzassi. Writing – review and editing: P. Makouloutou‐Nzassi, G. Ndong Atome and N.M. Longo‐Pendy. Visualisation: P. Makouloutou‐Nzassi, S.L. Oyegue‐Liabagui and N.B. N'dilimabaka. Supervision: P. Makouloutou‐Nzassi and G. Ndong Atome. Project administration: P. Makouloutou‐Nzassi and S.L. Oyegue‐Liabagui. Funding acquisition: J.B. Lekana‐Douki and S.L. Oyegue‐Liabagui. All authors have read and agreed to the published version of the manuscript.

## Funding

CIRMF is supported by Gabonese government via PID/PIH by Total Energies Gabon. CIRMF is a member of CANTAM funded by EDCTP [1045 CANTAM2 EDCTP ReNet2015].

## Ethics Statement

This study was approved by the National Research Ethics Committee of Gabon on 06 March 2018 (N001/PR/SG/CNER/2018), in accordance with the principles of the Declaration of Helsinki and relevant ethical guidelines. Participation in the study was voluntary, and informed consent was obtained from parents or legal guardians of children. All animal samples were collected from animals found dead. Thus, no ethical approval was necessary.

## Consent

The authors have nothing to report.

## Conflicts of Interest

The authors declare no conflicts of interest.

## Data Availability

The data that support the findings of this study are available from the corresponding author upon reasonable request.
